# Changes in Oxidative Stress, Inflammatory Markers, and Lipid Profile After a 6-Week High-Antioxidant-Capacity Dietary Intervention in CVD Patients

**DOI:** 10.3390/nu17050806

**Published:** 2025-02-26

**Authors:** Magdalena Czlapka-Matyasik, Lidia Wadolowska, Paweł Gut, Anna Gramza-Michałowska

**Affiliations:** 1 Department of Human Nutrition and Dietetics, Poznan University of Life Sciences, 60-624 Poznan, Poland; 2 Department of Human Nutrition, University of Warmia and Mazury in Olsztyn, 10-718 Olsztyn, Poland; lidia.wadolowska@uwm.edu.pl; 3 Department of Endocrinology, Metabolism, and Internal Medicine, Poznan University of Medical Sciences, 60-355 Poznan, Poland; gutpj@poczta.onet.pl; 4 Department of Gastronomy Sciences and Functional Foods, Poznan University of Life Sciences, 60-624 Poznan, Poland; anna.gramza@up.poznan.pl

**Keywords:** cardiovascular disease, dietary antioxidant capacity, dietary intervention, oxidative stress, urinary isoprostanes, oxidised low-density lipoproteins, α-tocopherol, β-carotene

## Abstract

**Background/Objectives**: Increased dietary antioxidant capacity is a good means of lowering oxidative stress and cardiovascular risk. Established antioxidant capacity doses should be tested using dietary intervention. **Methods**: We analysed the influence of a high-antioxidant-capacity diet on oxidative stress (OS) and inflammatory and lipid profile in CVD (cardiovascular disease) subjects with initially low (LowA) and high (HighA) antioxidant capacity markers. It was an experimental study with a 6-week dietary intervention (DI). Forty-eight CVD patients completed the DI. Blood and urine samples were collected, and anthropometric measurements were taken. Dietary data were collected using a multi-day food record method. α-tocopherol, β-carotene, and retinol were chosen as antioxidant capacity markers; F2-isoprostanes (F2-IsoP), oxidised low-density lipoproteins (oxLDL), and uric acid (UA) were used as OS markers; and interleukin 6 (IL-6) and high-sensitivity *C*-reactive proteins (hs-CRP) were used as inflammatory markers. Total cholesterol, low- and high-density lipoproteins, and triglycerides (TCHOL, LDL, HDL, TRI) as lipid profiles were analysed. Two groups of subjects with LowA and HighA profiles were identified. **Results**: The total dietary antioxidant capacity intake during DI was increased by 56%. In the total sample, the DI increased β-carotene, retinol, and UA, and decreased IL-6 oxLDL. The LowA group exhibited increased β-carotene, α-tocopherol, retinol, and decreased IL-6. The HighA group exhibited increased β-carotene and decreased IL-6, F2-IsoP, oxLDL, and oxLDL/LDL ratio. In the HighA group, compared to the LowA group, greater decreases in α-tocopherol and F2-IsoP were found. In both groups, inflammatory markers (IL-6) decreased, and β-carotene increased. **Conclusions**: The DI results depended on the antioxidant capacity profile at baseline; nevertheless, the established DI including selected antioxidative snacks significantly decrease oxidative stress and improve antioxidant capacity. Further research on diet natural antioxidant supplementation needs to be continued.

## 1. Introduction

Cardiovascular diseases have remained the leading cause of mortality for over three decades, amounting to approximately one-third of all global deaths, with 20.5 million deaths recorded in 2021 [1,2]. The Polish statistics confirm these data, since 28% of all deaths in 2021 were related to CVD [3]. The aetiology and mechanism of atherogenesis are still being investigated and discussed. Oxidative stress (OS), the imbalance between antioxidants and pro-oxidants, has been linked to CVD in many papers [1,4,5,6,7,8,9,10]. Much is already known about the molecular mechanisms of oxidative stress that lead to CVD. Its mechanism has been explored, with studies showing that endothelial damage and reactive oxygen species (and other free radicals) have predominantly emerged as factors in almost all paths leading to the development of atherosclerosis due to hyperlipidaemia, diabetes, hypertension, smoking, or an imbalanced diet with low antioxidant capacity [11,12]. Increased dietary antioxidant capacity is described in the literature as supporting the body’s endogenous enzymatic and non-enzymatic antioxidant defence systems, counterbalancing the adverse effects of oxidative damage due to free radicals [13,14,15,16,17]. According to these data, the role of diet composition, as one of many factors in preventing and treating CVD, cannot be overestimated, especially since 44.03% of CVDs in Poland (2017) could be attributed to dietary factors [3]. Generally speaking, diet is still the unquestionable leader in CVD prevention and diet therapy. However, its parameters are still being refined.

Despite many studies reporting the role of dietary antioxidant capacity as a factor limiting OS, the effect of a diet rich in natural antioxidants has yet to be studied extensively. The optimum dose of dietary antioxidant capacity to decrease OS, inflammation, and lipid profile still needs to be identified. From the PREDIMED studies, we know that there is an inverse association between changes in polyphenol intake and inflammatory status [18]. Similarly, in EPIC studies, it was found that higher inflammation and lower concentrations of the antioxidant nutrients β-carotene and vitamins A, C, and E were consistently significantly associated with higher odds of having multiple long-term conditions like myocardial infarction, stroke, type 2 diabetes, cancer, asthma, depression, arthritis, osteoporosis, hypertension, and obesity [19].

It is necessary to underline that changes in dietary markers due to high antioxidant dietary intervention (DI) related to its initial concentration have not been defined or evaluated. It has been shown that increased antioxidant capacity and decreased OS in the human body could decrease inflammation, CVD, and cancer risk [20,21]. Many epidemiological studies highlight the association between the consumption of polyphenols and CVD risk [22,23,24]. Some studies have examined the links between green tea consumption and reduced risk of CVD, especially in the Chinese population [25,26,27,28]. Various studies have proven the relationship between selected foods like grapes, raisins, and alcohol and OS changes [13,14,29,30,31,32]. Also, our previous preliminary studies analysing the inclusion of high-antioxidant snacks in the diet showed promising changes in subjects with cardiovascular burden and improved antioxidant body capacity [33].

It is worth underlining that studies on dietary antioxidant capacity were mainly conducted on Mediterranean, Chinese, or Spanish populations [15]. Data linked to Central European countries are limited [34]. For a while, studies also considered using supplements as an alternative to a diet containing natural food with high antioxidant capacity. However, this position has been verified by subsequent studies. The literature describes how the Carotene and Retinol Efficacy Lung Cancer Chemoprevention Trial (CARET) with supplements was terminated after unexpected findings, namely, that the active treatment group who were given a combination of 30 mg beta-carotene and 25,000 IU retinyl palmitate had a 46% increased lung cancer mortality risk and a 26% increased cardiovascular mortality risk compared with those given a placebo [35]. These results clearly outline the potential effects of overdosing on supplements and indicate that nutritional prophylaxis is a much safer alternative. As oxidative stress still represents a therapeutic target in CVD, the antioxidant capacity of the diet, its analysis, its potential to regulate, and its effects on the organism remain the main focus of interest, yet the clarification of doses, units, forms, and formulations is still of scientific interest. Studies have recognised the total supply of polyphenols as a significant antioxidant group in preventing CVD or overall mortality in different groups. Although not always unequivocal, such evidence was provided by EPIC and PREDIMED studies [36,37,38]. Higher polyphenol intake was associated with a lower incidence of overall mortality, blood pressure, inflammatory biomarkers, the onset of new type 2 diabetes mellitus (T2DM), and obesity [39,40,41].

In none of the observational cross-sectional studies described above did the authors provide information on antioxidant capacity and its changes under the influence of the intake of specific doses of natural antioxidants. Also, markers of antioxidant status, inflammation, or lipid metabolism were not always available. To date, markers of antioxidant capacity have not been clustered, and a holistic approach identifying an antioxidant capacity profile has yet to be considered during a controlled dietary intervention. More information must be recorded on the level of changes in OS, inflammation, and lipid profile concerning the defined antioxidant capacity of the diet.

Therefore, we designed a dietary intervention that monitored antioxidant capacity, OS, inflammatory markers, and lipid profile. Our study aimed to determine the influence of the high-antioxidant-capacity diet on OS and inflammatory and lipid profile markers in cardiovascular subjects with initially low or high antioxidant capacity markers.

## 2. Study Sample and Methods

The Bioethics Committee of the Poznan University of Medical Sciences approved the study protocol (Resolution no. 584/2011). Informed consent was obtained from the participants.

### 2.1. Study Design and Sample

**Figure 1:**
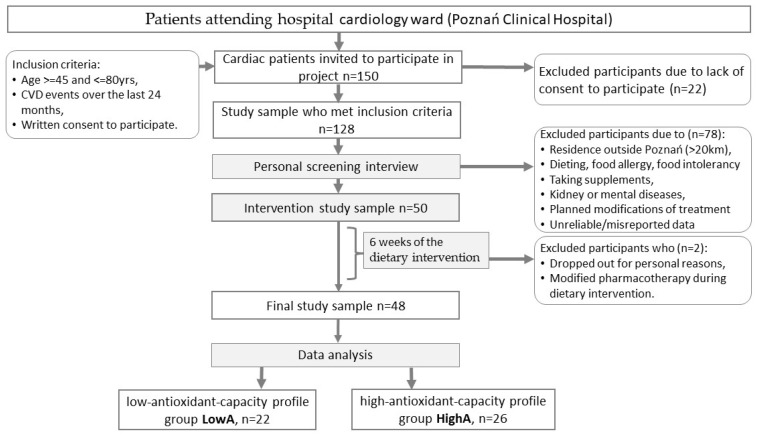
Patient flowchart.

This was an experimental study with a dietary intervention (DI) lasting 6 weeks. The sample was recruited in the clinical cardiac intensive care ward during hospitalisation (Figure 1). The inclusion criteria were 45 to 80 years, and cardiovascular events included a myocardial infarction over the last 24 months before hospitalisation. The exclusion criteria were a failure to meet the inclusion criteria, residence outside the Poznań area (>20 km), any dieting including weight reduction therapies, food allergy or food intolerance, kidney or mental diseases, planned modifications of pharmacotherapy in the course of the DI, and the consumption of dietary supplements (Figure 1). Initially, 150 cardiovascular patients were invited by medical staff to attend. The planned intervention programme was described in detail to all invited subjects during the individual interview. The course and plans of pending treatment modifications, pharmacotherapy, confirmation of concomitant diseases, and any dietary modifications were excluded. The place of residence was important due to the regular delivery of antioxidant snacks and requirement for personal contact with the subjects.

After the exclusion of 100 subjects, 50 started the DI. Finally, 48 cardiovascular patients (21 men, 27 women) aged 59.7 years on average completed the DI. The participants included in the study received no monetary compensation.

For further analysis, using accomplished data on standardised baseline plasma α-tocopherol, β-carotene, and retinol levels, a k-means clustering algorithm was applied to identify groups of individuals with initially low antioxidant capacity (LowA, n = 22) and initially high antioxidant capacity (HighA, n = 26). Details of the procedure are presented in Section 2.5 (Supplementary Table S1).

The sample size for the DI was calculated in regard to dietary antioxidant capacity expressed as a total Oxygen Radical Absorbance Capacity (ORAC) per 1000 kcal/person/day and on the expected change in this ratio in cardiovascular subjects after the DI. With a 5% significance level and 80% power, the minimal sample size required 40 subjects at the end of the DI to detect a 40% increase in total ORAC after the DI compared to the baseline. The adequacy of the sample size related to this analysis was checked for the data under study, and the post hoc statistical power was calculated. When comparing differences between two groups after DI (22 vs. 26) for urine F2-IsoP and plasma concentrations of oxLDL, uric acid, β-carotene, retinol, and α-tocopherol, assuming a 5% significance level, the statistical power was 7.3%, 2.9%, 97.8%, 100%, 30.5%, and 5.0%, respectively. Thus, we found that the sample size was sufficient to detect differences between groups if they existed.

### 2.2. Data Collection and the 6-Week Dietary Intervention

**Figure 2:**
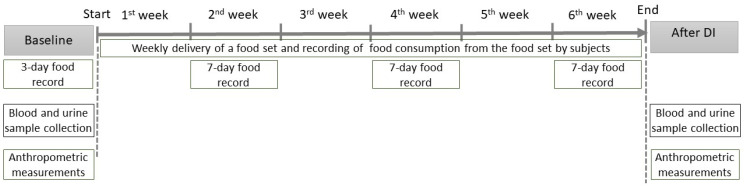
Timetable of dietary intervention and data collection. DI—dietary intervention.

The timetable of the dietary intervention and data collection procedures are shown in Figure 2. Data concerning demographic variables, age, smoking, education, current health status, medical history, and pharmacotherapy were collected via self-administered questionnaires with closed-ended multiple-choice questions during the initial screening interview at the hospital and were confirmed before the DI. A well-trained medical staff member and dietician interweaved subjects at the hospital and during the DI.

Dietary data were collected using a multi-day food record method: a 3-day food record at baseline, and three 7-day food records during the 2nd, 4th, and 6th weeks of the DI. Blood and urine samples and anthropometrics were collected at baseline and after 6 weeks of the DI. Biological material was protected and stored at a certified laboratory. Anthropometric measurements were taken by a trained dietician (Figure 2).

### 2.3. Dietary Intake at Dietary Intervention

Before DI, subjects received counselling to follow usual, habitual dietary for the whole dietary intervention. Besides their usual diet, the subjects received a weekly delivery of a high-antioxidant-capacity “food set” and kept a record of the total daily intake (Supplementary Table S2; Figure 2). We proposed a set of foods with increased antioxidant potential based on our previous experience; the aim behind the selection of products was acceptability and popularity in this group [34]. The food set contained quantified items of 90% dark chocolate, strawberry crisps, red dry wine, dried cranberries, 100% chokeberry–apple juice, dried oregano, thyme, basil, dill, walnuts, hazelnuts, almond, grated beetroot with horseradish, prunes, beetroot crisps, green tea, dried apricots, black pepper, apple crisps, 100% tomato juice, tomato crisps, and olive oil. Detailed information on the amounts of high-antioxidant foods consumed by the participants during the DI is presented in Supplementary Materials. The respondents were asked to include these foods in their habitual diet. Additionally, the dieticians prepared recipes and information about the beneficial health properties of the foods included in the food set to increase dietary compliance and encourage the subjects to use the set of products in their daily diet. This information was included weekly and delivered to the subjects with the food set.

The dietary recordings obtained at baseline; in the 2nd, 4th, and 6th week of the DI; and from the records of daily “food set” intake were collected and analysed every week by a trained dietician, as recommended in the literature and our previous studies [42,43]. To ensure the quantity and quality of the consumed food set, the subjects were asked to collect empty food packaging after consuming items from the set. The amount of food was determined using an “album of photographs of food products and dishes” and expressed in grams [44].

The antioxidant capacity of the food set and total dietary intake were calculated in terms of total Oxygen Radical Absorbance Capacity (ORAC) and expressed in μmol of Trolox Equivalent/person/day (μmolTE/day). The ORAC describes the total capacity of the mix of antioxidants contained in food to inhibit free oxygen radicals and was calculated based on the USDA ORAC database of selected foods [45,46,47]. The total ORAC of the food set intake was 18,531 μmolTE/day on average, while the ORAC of the total daily intake was 22,674 μmolTE/day on average (23,991 vs. 21,560 μmolTE/day, LowA vs. HighA groups).

The mean daily energy and selected nutrient intake were calculated for each respondent using “Energia v.4.1” software with an implemented food database. The food database was composed of 962 items from the official database of foods commonly consumed in Poland [48] and manual inputs concerning ORAC (from the USDA database [47]). Plate waste was estimated using the software’s built-in options and accounted for 10% of the energy and all of the macro- and micronutrients (10%), except for vitamin C (55%), folic acid (40%), vitamin A (25%), B1 (20%), B2 (15%), and niacin (15%). Other supplements that could have antioxidant effects were prohibited during the intervention period. The pharmacotherapy prescribed and followed was classified according to the Anatomical Therapeutic Chemical Classification System (ATC) (Table 1). The total dietary records before the DI (baseline) and during the 6-week DI were completed, averaged, and compared.

### 2.4. Assay Methods

The urine 15-isoprostanes F_2t_ isoprostanes (F2-IsoP, ng/mL, and ng/mg creatinine), serum uric acid (UA, mg/dL), and oxidised low-density lipoprotein (oxLDL, µg/mL) were selected as an oxidative stress marker, while α-tocopherol, β-carotene, and retinol (all in µg/mL serum) were used as antioxidant capacity markers. Serum IL-6 (pg/mL), high-sensitivity *C*-reactive protein (Hs-CRP, pg/mL), and creatinine (mg/dL) were inflammatory markers. Blood samples were collected by a registered nurse after an overnight fast at baseline and at the end of the DI for biochemical assessments.

The blood samples were drawn from the antecubital vein of the subjects in the morning. Laboratory tests for complete lipids and hsCRP were performed on the day of the blood collection. For other tests performed later, plasma or serum was stored immediately after retrieval at −70 °C.

The metabolic variables of triglycerides, total cholesterol (TC), high-density lipoprotein (HDL) cholesterol, and low-density lipoprotein (LDL) cholesterol were determined from serum using routine biochemical methods (all Cobas, Roche, Penzberg, Germany). Serum hs-CRP (mg/L) was measured by the immunoturbidimetric method described previously [49].

F2-IsoP was measured using a competitive enzyme-linked immunoassay (ELISA) (Neogen Corporation, Lexington, KY, USA) with the absorbance of the reader set at 650 nm. Each sample was analysed in duplicate [50]. The concentration of F2-IsoP in urine was adjusted for urinary creatinine to account for different urine diluteness between individuals, which varies depending on how much liquid a person ingested and how much was reabsorbed in the kidneys [51]. The oxidised low-density lipoproteins (OxLDLs) were measured by a commercially available solid-phase, two-site, enzyme immunoassay sandwich ELISA (Biomedica, Uppsala, Sweden, NR BI20042) [52,53]. The plasma levels of uric acid (UA) (mg/dL) were determined using the uricase–peroxidase method [54].

The determination of plasma α-tocopherol, β-carotene, and retinol was based on standard methods [55,56,57] using HPLC. Plasma was obtained by centrifugation (1000× *g* for 15 min at room temperature), and then stored in the dark at −80 °C until analysis. Extractions were performed in duplicate. One millilitre of plasma was deproteinised by the same volume of ethyl alcohol containing internal standards, and lipophilic components were extracted twice by adding two volumes of *n*-hexane. The mixture was vortexed for 30 s and then centrifuged at 1000× *g* for 5 min at room temperature. Both hexane phases were collected and evaporated under nitrogen. The dry residue was dissolved in 200 μL of acetonitrile–dichloromethane–methanol (75–10–15) and transferred into a 2 mL glass screw-top vial for automatic sampling, using 40 μL for HPLC. The HPLC was run on a Waters Alliance system. β-carotene, retinol, and α-tocopherol were detected at 450, 325, and 292 nm, respectively, and identified by comparing the retention times and spectral analyses with those of pure standards [57].

IL-6 (pg/mL) was assessed using commercial ELISA kits [58,59,60] using an immunoenzymatic test (Human IL6 ELISA Kit for Serum, Plasma, Cell Culture Supernatant and Urine RayBiotech Life, Inc., Peachtree Corners, GA, USA) [58,60].

Serum hs-CRP (mg/L) concentrations were measured using a highly sensitive immunoassay [49]. Concentrations were evaluated using a modification of the Behring latex-enhanced CRP assay on a BN-100 nephelometer (Dade Behring, Newark, DE, USA) with a 2% intraassay coefficient of variation [61].

### 2.5. Statistical Analysis

Primary analyses with paired *t*-tests were performed to compare biochemical markers and dietary intake in the total group before and after the intervention. Secondary analyses aimed to evaluate the influence of baseline plasma antioxidant capacity on intervention compliance. Using data on standardised baseline plasma α-tocopherol, β-carotene, and retinol levels, a k-means clustering algorithm was applied to identify groups of LowA and HighA individuals (Table 1; Supplementary Table S1). Two criteria determined the number of clusters: (1) hierarchical Ward clustering algorithm and (2) cluster size > 10% of the total sample size. Optimal clustering was found for a 2-cluster solution, which divided the initial sample into a low-antioxidant-capacity group (“LowA”, n = 22) and a high-antioxidant-capacity group (“HighA”, n = 26). Linear mixed-effect models were used to compare the outcome variables across two time points (week 0 vs. week 6) between groups in terms of antioxidant capacity. The interaction term Time*Group was included in the model to compare the over-time change between the two groups. The models were adjusted for sex as a binary variable and age, BMI, energy intake, and ORAC (µM Trolox per 1000 kcal) as continuous variables. Tukey–Kramer post hoc tests were applied to test for multiple comparisons.

The sample characteristics were summarised as the mean with standard deviation or a number with percentage differences in characteristics between antioxidant capacity groups, and were tested using the Chi-square or Fisher’s exact test for categorical variables and the *t*-test for continuous variables. The assumption of data normality was assessed using histograms and QQ plots. Skewed variables were logarithmically transformed to ensure the normality of distribution. The outcome of interest variables were presented as a mean with a 95% confidence interval (CI) or, if non-normally distributed, as a geometric mean with a 95% CI.

All calculations were performed using R (version R 3.4.3; R Foundation for Statistical Computing, Vienna, Austria) with nlme, lsemans, and cluster packages. *p*-values < 0.05 were considered statistically significant for all analyses.

## 3. Results

### 3.1. Sample Characteristics

The main characteristics of the study sample, including the categorisation of LowA and HighA participants, are summarised in Table 1. The mean age of the subjects was 59.7 years. The average BMI was 30.5 kg/m^2^, and 44% of the subjects had android-type obesity (waist 105 cm). Almost one-third (27%) of subjects were current smokers. Most subjects (88%) lived in urbanised areas, and 48% declared being physically active. As shown in Table 1, the study participants were diagnosed with hypertension (21%), hyperlipidaemia (58%), and diabetes mellitus (23%). The subjects were treated with antithrombotic agents (8%), BP-lowering (8%), and lipid-modifying (8%) drugs. No differences between the LowA and HighA groups were found regarding age, BMI, waist, socioeconomic variables, treatment, and diagnosis (Table 1).

### 3.2. Changes in Oxidative Stress Markers

The mean values of the changes in blood and urinary biomarkers in the total sample and by low- and high-plasma-antioxidant-capacity groups are presented in Table 2.

The baseline average urine F2-IsoP level in the total sample was 2.62 ng/mL. However, in the HighA group, it was more than twice as high than in the LowA group. Moreover, the urine F2-IsoP in the HighA group decreased significantly during the DI (39%, *p* < 0.001). A significant difference (*p* < 0.01; 48%) between changes in the LowA vs. HighA group in urine F2-IsoP was observed.

The findings showed that the dietary intervention, on average, decreased oxLDL by 24% (*p* < 0.01) (Table 2). The initial level of oxLDL in the whole group was 1.11 µg/mL and did not differ between the HighA and LowA groups. Only the changes during the DI observed in both groups separately showed that, in the HighA group, the decrease in oxLDL was significant (27%, *p* < 0.01), while the difference in the change between groups was not significant (Table 2).

The initial UA level in the total sample was 5.6 mg/dL, which significantly (*p* < 0.05) increased during the DI by 6%. Simultaneously, there were no significant differences when comparing the changes in the LowA and HighA groups (Table 2).

### 3.3. Changes in Antioxidant Capacity Markers

Significant differences in antioxidative capacity markers, like α-tocopherol and retinol, between the LowA and HighA groups were observed at baseline (Table 2). There were no differences in β-carotene concentration.

The average α-tocopherol concentration in the LowA group was 12,4 μg/mL, while in the HighA group it was 17.0 μg/mL (Table 2). The findings showed that DI in the LowA group resulted in a 17% (*p* < 0.001) increase in the concentration of α-tocopherol, while the differences between groups in terms of the changes in α-tocopherol concentration were found to be 131% (*p* < 0.001) in favour of the LowA group. The mean level of β-carotene in the total sample was 0.96 μg/mL and increased (*p* < 0.001) in concentration after the DI in both the LowA and HighA groups by about 23 vs. 22%, respectively. The retinol concentration in the total sample at baseline was elevated at 2.7 μg/mL (LowA: 2.39; HighA: 2.89). A significant increase of 11% (*p* < 0.001) was noted in the LowA group and was higher than in the HighA group by 56%.

### 3.4. Changes in Inflammatory Markers

The findings in Table 2 show a significant decrease in inflammation marked by IL-6. The initial level of IL-6 (4.6 pg/mL) in the total sample significantly (*p* < 0.001) decreased by 50%. This reduction was confirmed in the LowA and HighA groups (40%, *p* < 0.05 vs. 50%, *p* < 0.001) separately. There were no significant changes during the DI in terms of the other inflammatory markers, such as hs-CRP. Nevertheless, we found high variability for this parameter when we examined individual changes.

### 3.5. Changes in Lipid Profile

No significant changes in lipid profiles like HDL, HDL/LDL ratio, TC, or TRI were noted for any of the studied groups (Table 2). However, a significant (*p* < 0.05) improvement (−19%) in the OxLDL/LDL ratio was observed in the total sample and was confirmed in the HighA group (−24%, *p* < 0.05).

### 3.6. Dietary Intake During Dietary Intervention

The total dietary intake and changes during the dietary intervention are presented in Table 3. The indexes of the antioxidant capacity of the food set delivered during the DI are also presented in Table 3.

The total dietary antioxidant capacity intake of the whole study sample before the DI was 22,674 μmolTE/day and increased during the DI by 56% (*p* < 0.001) due to the subjects’ consumption of the high-antioxidant-capacity snacks delivered to them weekly. The indexes of the antioxidant capacity of the food set delivered to the participants are presented in Supplementary Table S2. On average, 18,529 μmolTE/day was offered to the subjects. The utilisation percentage of the weekly antioxidant snacks for daily consumption was 47%.

The analysis of the changes in antioxidant density (Q-ORAC) during the DI found a 68% (*p* < 0.001) increase in Q-ORAC from 13,760 µmolTE/1000 kcal in the total sample, a 58% (*p* < 0.001) increase in the LowA group, and a 77% (*p* < 0.001) increase in the HighA group.

Additionally, during the 6 weeks of the DI, an 8% increase in total carbohydrates was observed across the whole study sample. There was almost no increase in the LowA group, unlike in the HighA group, where a 15% (*p* < 0.001) increase was noted.

During the DI, the structure of the fats consumed changed. The total sample exhibited a decrease in cholesterol and SFA by 15% and 20% (*p* < 0.01), respectively.

## 4. Discussion

This research represents the initial exploration of the connections between a monitored 6-week high-antioxidant-capacity dietary intervention and changes in oxidative stress, body antioxidant capacity, inflammatory markers, and lipid profiles in a group of CVD patients after cardiovascular events. The changes in antioxidant capacity we describe have not yet been widely published as a whole-controlled nutritional intervention.

Several findings from our study were noteworthy. Primarily, what distinguished our study from previous studies is that it was an observational study conducted in real-world settings. Overall, we have shown that it is possible to modify the body’s antioxidant capacity using dietary profiling; nevertheless, the magnitude of this change depends on its baseline values. Our study showed a 56% increase in dietary antioxidant capacity and an average 24% decrease in oxidative stress levels, as measured by oxLDL concentrations. At the same time, we observed an increase in the body’s antioxidant capacity expressed by an increase in β-carotene (23%) and retinol (7%) and a significant (50%) decrease in the inflammatory marker IL-6. The least remarkable changes were observed in the lipid profile, the most common tool for monitoring cardiovascular disease (CVD) risk. Somewhat surprising is the lack of changes in the lipid profile of the two groups, although we think that this may be related to the small sample size, especially since we observed no statistically significant trends when analysing the results. The fact that this was an interventional study, providing one of the most substantial pieces of scientific evidence, adds further value to the research presented.

Our findings align with many studies that emphasise the protective effects of increased antioxidant capacity as a diet parameter. For instance, a systematic review by Aleksandrova et al. (2015) showed an inverse association between the Mediterranean Dietary Approaches to Stop Hypertension (DASH) diets, the vegetarian diet, the USDA Healthy Eating Index (HEI)-based diet, and the palaeolithic diet and oxidative stress and proinflammatory biomarkers [62]. Positive associations between OS and inflammatory markers were revealed for Western and fast-food diets [62]. Koelman and Estruch et al. showed that the Mediterranean diet was the most prominent reducer of inflammatory biomarkers (IL-6, IL-1β, and *C*-reactive protein) [20,63]. Other authors confirmed the beneficial effect of the increased dietary antioxidant activity on oxidative stress, inflammation, and *C*-reactive protein [63,64,65,66,67]. Some authors suggest that the CVD risk is lower in groups whose diets have higher total antioxidant capacity [65,66].

What distinguished our study from others is the quantitatively monitored intervention, prior to which we determined the habitual antioxidant capacity of the CVD subjects’ diets, and then included a selection of free-choice snacks with increased antioxidant density.

There is limited information in the literature about the level of dietary antioxidant capacity in different populations. The following are known published U.S. data, ranging from 4600 to 7400 units of ORAC per day [67,68,69]. In the Singapore Health Study, the dietary ORAC intake was 5600 daily [70,71]. The Swedish authors mentioned above found wide diversity and analysed the dietary antioxidant capacity between the first and fourth quartiles, finding values from 7670 to 18,370 ORACs/daily. Noteworthy is that they found a nearly 50% lower risk of stroke in women with a dietary antioxidant capacity score in the three highest quartiles. Rautiainen et al. concluded that a greater risk of haemorrhagic stroke was observed with the consumption of less than 7400 ORAC units per day [72].

Our previous studies revealed that CVD patients had significant seasonal variability in dietary antioxidant capacity, ranging from 19,915 in spring to 30,815 ORACs in summer [34]. Nevertheless, these data corresponded with our other data, where the dietary antioxidant capacity of women aged 25–40 stood at 18,661 ORACs [73]. Finally, we would like to emphasise that insufficient research data exist to establish reference dietary levels of total antioxidants (measured, for example, by Oxygen Radical Absorbance Capacity ORAC) [74,75]. However, a review of the existing literature and recent studies leads to the assumption that an ORAC of at least 12,000 units per day may provide a reduced risk of the diseases studied, while an intake of less than ~7000 ORAC units per day may lead to an increased risk of certain diseases. These recommendations align with a daily consumption of 7 to 10 servings of fruits/vegetables/grains, with some foods being higher in ORAC [74].

The average dietary antioxidant capacity (ORAC) revealed in our study was 22,674 μmolTE/day, which was slightly higher than in the study by Rautiainen et al. [72]. Our CVD group did not have such a long-proven CVD history, but the participants had suffered a cardiovascular event like stroke or myocardial infarction in the last 2 years, which may have influenced a more remarkable improvement in habits, nutrition, and health care during the recovery period.

In our experience, an exemplary method of modifying eating habits is offering patients alternative foods and not prohibiting the consumption of unfavourable foods. Convenience is one of the most emphasised arguments for changing dietary habits [76,77]. Therefore, based on previous studies, we designed a set of foods popular with this group that were acceptable and convenient for preparation, and delivered weekly [33,34,73]. The food set offered weekly to the subjects for consumption during the 6-week dietary intervention included dark chocolate (90% cocoa), strawberry, apple, carrot, tomato, beetroot crisps, red wine (*cabernet sauvignon*), dried cranberries, prunes, apricots, chokeberry–apple juice (100%), oregano, dill, thyme, basil, pepper, walnuts, hazelnuts, almonds, grated beetroot with horseradish, tomato juice (100%), olive oil, and green tea. In order to check the amounts consumed, we carried out detailed monitoring of consumption (food records) and asked the subjects to keep and return empty packaging. The total antioxidant capacity of the weekly delivered set was 18,529 μmolTE/day, 47% of which was consumed. These consumed snacks were included in the analysis of the daily antioxidant capacity.

The body’s antioxidant capacity is crucial for maintaining cellular homeostasis and preventing oxidative damage, which can contribute to various diseases, including cancer, cardiovascular disease, and neurodegenerative disorders [78]. Oxidative stress refers to the pathological situation in which the excessive production of oxygen free radicals and antioxidant defence or repair capacity defects lead to an accumulation of oxygen free radicals and related metabolites, inducing cell toxicity. The excessive presence of ROS/RNS (reactive nitrogen species) or defects in antioxidant capacity could lead to protein, lipid, or DNA oxidation, and cells could be severely damaged [57,58,59]. These processes are invariably accompanied by inflammation. To monitor the DI, we used markers of these three states: oxidative stress, antioxidant capacity, and inflammation.

OxLDL, F2-IsoP, and UA were used to monitor oxidative stress. OxLDL, perceived as an oxidative stress marker in CVDs, has been widely confirmed [79,80]. A favourable decrease in oxLDL was observed in all subjects in the DI group by an average of 24%. These changes were confirmed and were more pronounced in the group with initially higher antioxidant capacity (HighA: 27%). However, quantitative estimates of the dietary modification associated with a quantified increase in the total dietary antioxidant capacity and its effect on the modification of oxidative stress markers are unknown. The literature describes a reduction in oxLDL (by 8.2%) during an 8-week nutritional intervention [31]. The authors observed the aforementioned change after the participants consumed 200 mL of grapefruit juice daily. However, the study did not report the degree of dietary modification of the subjects concerning the pre-intervention period, nor did it analyse the total nutritional value of the diet during the intervention. Our results align with a Spanish experiment in which Martinez-Tomás et al. (2012) showed an 8.2% decrease in ox-LDL concentrations during a 4-week intervention involving the consumption of a vegetable soup rich in natural antioxidants [81]. The Spanish study did not monitor ORAC levels during the intervention, making it impossible to compare the findings with the results of our study. A well-known study by Cao et al. also determined that the average antioxidant capacity (ORAC) from fruit and vegetables in subjects’ diets in the year preceding the experiment was 1420–1920 ORAC/day [82]. During the 15-day nutritional intervention, the authors administered approximately 3000 ORACs daily as fruits and vegetables; nevertheless, in this study, other sources of dietary antioxidants were not analysed, and the markers of oxidative stress in the body used were alpha-tocopherol and the ORAC index (as recommended for in vitro studies), which makes it impossible to relate the findings to those of our study.

In summarising the changes in oxLDL levels, as influenced by the DI, it can be concluded that in both the LowA and HighA groups, no significant changes were observed in the supply of antioxidant vitamins/provitamins (A, E, and C and beta-carotene) commonly considered to improve the antioxidant capacity of the diet. This may suggest that the reduction in OS may be related to changes in the levels and structure of total fats and carbohydrates, as well as non-nutrients such as polyphenols and flavonoids.

This hypothesis is reinforced by the study of Tesoriere et al. (2004), who showed, during a DI, that the natural antioxidants present in fresh fruit pulp had a more significant effect on improving antioxidant status than vitamin C as a supplement [83].

F2-isoprostanes are a biomarker of lipid peroxidation and are recognised as a good marker of oxidative stress in cardiovascular diseases, and have, therefore, been extensively examined and validated [84,85]. The changes in isoprostane concentrations in this project were already less pronounced during DI than other markers. Interestingly, we observed a 39% decrease in concentration in the HighA group after the 6-week DI, while in the LowA group, on the contrary, the increase was not statistically significant.

However, the analysis of isoprostane concentrations highlights two aspects. Their baseline concentrations differed before the intervention and were significantly higher in the HighA group, hence the more remarkable change in this group. In addition, the HighA group changed their diet much more regarding the amount and type of fat intake.

Numerous studies have examined isoprostane changes during dietary interventions. Our results follow those published by Meyer et al., who revealed a negative association between F_2_-isoprostanes and diet quality score and the fruit–veg diet pattern, and a positive association between F_2_-isoprostanes and the meat diet pattern [86].

Interesting results were found on the influence of coffee intake on isoprostane changes during four- and eight-week regular consumption. In the first study by Kempf et al., 47 volunteers with an elevated risk of type 2 diabetes were sequentially given two different doses of coffee (four and eight cups/day) for four weeks each, leading to a significant decrease in the serum concentration of isoprostanes for the eight cups/day dose [87]. In the second study, 8 weeks of administration of 184 mL/day of hydroxyhydroquinone (HHQ)-reduced coffee (containing 300 mg of chlorogenic acids) to nine subjects with mild hypertension and vascular failure led to a significant decrease of urinary isoprostanes [88], providing evidence for dietary antioxidants’ ability to successfully modulate isoprostane levels. In their literature review, Petrosino and Serafini (2014) confirmed that the correspondence between the effect on isoprostane and non-enzymatic antioxidant capacity has been evidenced [89].

As the last marker of oxidative stress, we analysed uric acid. Theories on uric acid in the body and the pathogenesis of CVD are divided. UA is considered one of the most important markers of CVD risk, with higher levels implying an increase in the risk mentioned above [90,91,92]. As reported by other authors, uric acid is also one of the most important low-molecular-weight antioxidants in the physiological fluids of the body [93,94,95]. Its monitoring in CVD patients is recommended, as it is a sensitive marker of ongoing inflammation in serum, leading to atherosclerotic plaque formation [96]. The mean serum concentration of the subjects in our study was 5.6 mg/dL and increased by 6% during the DI. The changes in UA concentration reported in the literature and their interpretation are inconclusive. According to Hellsten et al. (1997), it can be used as an antioxidant under conditions of increased oxidative stress [97]. In addition to its free radical scavenging function, protection against the inactivation of superoxide dismutase is mentioned [94]. Uric acid can inhibit xanthine oxidase by acting as an enzyme inhibitor, and this effect is associated with an increase in the production of superoxide radicals [98]. However, it is indicated that the action of uric acid may be concentration- and environment-dependent. Under conditions of high oxidative stress and the depletion of other antioxidants, uric acid can become a pro-oxidant. Some authors say that this phenomenon can occur from concentrations > 4 mg/dL [99,100].

Thus, the increase in uric acid concentration during the dietary intervention can be interpreted as the appearance of other potent antioxidants in the body, which may have taken over the antioxidant functions. As a result, less uric acid is used as an antioxidant. Moreover, changes in its levels can be interpreted as a reduction in the severity of oxidative stress, bearing in mind that it can only be used as a complementary marker. Summarising the changes in UA levels during the intervention, it should be emphasised that the complex metabolism of uric acid and its antioxidant effect result from plasma concentrations, which consist of its production and utilisation in tissues [97,101,102].

In parallel with the reduction in oxidative stress, the markers of antioxidant capacity improved. In our study, the mean blood level of alpha-tocopherol in the study group (14.9 µg/mL serum, i.e., 34.60 µM) was similar to that found in patients with Parkinson’s disease [103], lower than that of healthy patients (47.5 µM) or patients with fatty liver (40.9 µM) in the study by Bahcecioglu et al., and similar to the low level (9.15 µg/mL) found by Greek researchers in a population-based study [103,104,105]. The literature also confirms that its reduced levels in the blood are associated with a high level of oxidative stress [106].

In the DI, an average increase in blood alpha-tocopherol levels of 4% in the whole study group and a significant increase of 17% in the group with the initially lower antioxidant capacity (LowA) was observed.

Changes in alpha-tocopherol levels may indicate the intrinsic homeostasis of this vitamin. The literature shows that tocopherol and retinol blood concentrations during prolonged oxidative stress may increase as they are released from the body’s reserves. Aguilo et al. (2005) described such a mechanism in athletes during cycling training [107]. Gornicka also highlighted the issue of alpha-tocopherol release from the liver under increased oxidative stress [108]. According to Rietjens et al. (2002), there is an increase in alpha-tocopherol under conditions of oxidative stress, which can generate higher concentrations of tocopheryl radicals and lipid peroxidation [109].

Exploring this issue, it is worth underlining that when the antioxidant system is balanced, other antioxidants, such as ascorbic acid, restore it to its native form. Alpha-tocopherol is an exogenous antioxidant that interferes with radical reactions by transferring hydrogen atoms or electrons to the radicals and forming stable chemical structures. This group of compounds includes phenols (gallates), hydroquinones, and the aforementioned tocopherols [110]. The reaction of alpha-tocopherol with lipid radicals produces tocopheryl radicals, which can be reduced to vitamin E with vitamin C [111].

Carotenoids are classified as exogenous antioxidants whose action, like many other antioxidants, is synergistic. They are capable of scavenging oxygen and chelating ions involved in radical formation. Their antioxidant activity can be mediated by a mechanism of addition, electron transfer, or the transfer of a hydrogen atom to a radical [112]. Carotenoids can take energy from the singlet molecule without damage, thus protecting cells from changes associated with, for example, ageing or diseases of old age [113,114,115,116]. The action of beta-carotene is the quenching of lipid peroxyl radicals, which is complementary to the antioxidant action of alpha-tocopherol. Several studies have confirmed the role of beta-carotene in regulating oxidative stress and preventing CVD [117,118,119,120].

The measurements for beta-carotene obtained in the present study (0.95 μg/mL serum) were higher in comparison to those obtained among subjects following the Western dietary model in a German study (0.0107 µg/mL) of patients with systemic inflammatory response syndrome (SIRS) (0.322 μg/mL), and in healthy elderly individuals (0.418 µg/mL) in whom reduced beta-carotene concentrations were explained by lesions [121,122,123].

During the 6-week dietary intervention, which was associated with an average increase of 56% in total dietary antioxidant capacity, there was an average 23% increase in serum beta-carotene, which was similar in both subgroups, regardless of the baseline levels of other oxidative stress markers. The reasons for the observed increase in blood concentrations of beta-carotene, despite no significant alterations in dietary intake of the subject group, remain unclear.

The phenomenon of mutual compensation can be posited as a potential explanation for the observed increase in the concentration and utilisation of another compound that exhibits antioxidant properties similar to or identical to those of the initial compound. This phenomenon occurs when the initial compound exhibits a deficiency or is being utilised. In a situation of increased oxidative stress, body reserves of this vitamin may also be released, which, as with retinol, may also have occurred here [124,125].

Moreover, it is important to acknowledge the presence of significant individual variability among the subjects, which rendered clear inference and regression analysis challenging. This is exemplified by the data presented in the tables.

During the dietary intervention, for the low-antioxidant-capacity (LowA) group, an increase in blood beta-carotene concentration was observed, accompanied by a significant increase in retinol (11%) and alpha-tocopherol (17%) and a decrease in IL-6 concentrations.

Similar attempts at dietary modification to increase beta-carotene concentrations were made by Martinez-Tomas et al. [81,126]. The authors administered a vegetable soup rich in natural antioxidants to healthy volunteers over 4 weeks, resulting in a 67% increase in serum beta-carotene concentrations. These results of improved dietary beta-carotene balance call into question the advisability of supplements, the effects of which may increase the risk of serious health complications if the dose and target group are not matched [127,128,129].

Another marker of antioxidant capacity whose concentration was monitored during the nutritional intervention was retinol. In addition to its role in vision processes, growth, development, and cell differentiation [111,130], its antioxidant function of scavenging superoxide free radicals and quenching singlet oxygen is known and has been described [131,132]. From a CVD perspective, an important function of vitamin A is to protect low-density lipoprotein (LDL) from oxidation and the atherogenic modification of apolipoprotein B [133].

In the present study, higher initial retinol concentrations (2.7 µg/mL or 9.29 µM) were reported in comparison to the values described in the literature for women with breast cancer (1.82 µM) [134]. In addition, these concentrations were observed to be lower in patients immediately following ischaemic stroke (1.66 µM) [135] and in healthy men (1.6 µM) [136]. According to Ruiz Rejon et al. (2002), who found a 20 per cent increase in plasma retinol levels one year after acute coronary syndrome in their subjects, higher levels of this vitamin may be related to homeostatic regulatory mechanisms and a health-promoting attitude in convalescents, which was also observed in the present study [137].

The present study showed a mean increase of 7.7% in the serum retinol concentration during the DI. A more detailed interpretation of the results reveals a significant increase in the concentration of retinol in the LowA group (LowA: 11% vs. HighA: 4%). In interpreting the changes in plasma retinol concentrations, it is important to acknowledge that lower levels are less prevalent in older individuals. Mercocci et al. observed that concentrations of vitamins A and E, compared to beta-carotene, are increased in people of around 100 years of age [138]. Among the hypotheses positing an explanation for this phenomenon, the authors cited the greater affinity of fat-soluble vitamins for longevity. They suggested that the role of vitamin A is related to its effect on immune processes at this age. In addition, changes in vitamin A levels can be determined by its metabolism and permeation across the blood–brain barrier in the presence of plasma proteins. Therefore, its concentration is determined by retinol-binding proteins, which regulate its plasma levels [139]. Retinol-binding protein levels were not analysed in this study. One factor that may influence plasma retinol levels is the seasonality of consumption and the resulting changes in blood concentrations [140].

In conclusion, our study achieved the goal of determining the influence of a high-antioxidant-capacity diet on OS and inflammatory and lipid profile markers in CVD subjects. We are aware that while our study represents a significant step forward in research on improving the oxidoreductive balance in the body, it is crucial to acknowledge certain limitations.

Although this was a tightly controlled intervention with total dietary intake being closely monitored, the number of subjects was limited, and the possibility of inference by gender was restricted. As a variable, it would also be valuable to assess the subjects’ habitual diet before the CVD event.

Referring to the sample size and limited intervention time, we recruited 150 CVD subjects. However, the final sample, due to planned therapy modifications, cardiac procedures, and logistic limitations (place of living preventing frequent contact during intervention) was limited to 48 subjects (male and female).

The duration of the dietary intervention was chosen based on the literature; however, a discussion of the intervention’s duration may raise numerous questions. It is acknowledged that specific markers, such as the lipid profile, may require additional time.

Further, gender diversity could have influenced the results. In the LowA group, 36% of men participated, while the HighA group included 50% men. Since the study sample was limited, we did not divide the groups by gender. Nevertheless, as shown in our previous project, there were minor differences between men and women in terms of dietary preferences concerning sources of antioxidants in this group [34].

Finally, the markers of oxidative stress and antioxidant capacity were chosen based on the literature review, the authors’ experience, and financial limitations. During the DI, we did not monitor the levels of enzymes such as catalase (CAT), superoxide dismutase (SOD), or glutathione peroxidase (GPX). The objective of the present study was to concentrate on markers directly related to the mechanism of cardiovascular disease. However, it is acknowledged that their analysis has the potential to broaden the inferences made.

Most of the markers were analysed using ELISA, which remains a popular method in diagnostics due to its versatility and ability to detect low concentrations of analytes. In spite of its advantages, the method has some downsides since it requires precision and skill as other substances in the sample can interfere with the results. Our data were analysed in certified laboratory by a technician specialised in oxidative stress analysis.

In subsequent studies of dietary antioxidant capacity, we plan to focus on the seasonality and structure of dietary antioxidant capacity (i.e., fat-soluble and water-soluble antioxidants) and their categories (e.g., flavonoids, flavones, proanthocyanidins). These have been included in the table with the dietary assessment of this project. However, it should be noted that an appropriate indication of the levels was not available for all of the products consumed by the subjects, so interpretations must be made with caution.

## 5. Conclusions

In many ways, the control of oxidative stress and changes in the body’s antioxidant capacity represent a biochemical, logistical, and methodological puzzle. Nevertheless, the present study indicates that the monitoring of these factors reveals some rules of thumb that can be used to predict the direction of beneficial changes. In conclusion, our data show that changing the diet’s antioxidant capacity is undeniably important in profiling the body’s antioxidant capacity and oxidative stress markers in CVD patients. Firstly, we have shown that an average increase in the dietary antioxidant capacity of 56% relative to the baseline dietary capacity is possible; using natural food products, not supplements, is particularly interesting in this context. Secondly, we proved the effectiveness of this change and showed that it can induce beneficial changes in the body’s oxidative stress and its potential ability to counteract its effects.

Additional interesting information was provided by dividing the study group into individuals with initially low and high antioxidant capacity of the body. Here, the results indicated that the changes in antioxidant capacity and oxidative stress were dependent on baseline values. In the LowA group, during the DI, the antioxidant capacity increased by 58%. Dietary changes in HighA subjects were also associated with a significant increase in total carbohydrates and a decrease in SFA, which may further indicate that overall diet quality further supports changes in the antioxidant capacity of the subjects.

Our findings suggest that whole-diet ingredients should be considered when addressing the role of dietary antioxidants in health. Furthermore, the simultaneous evaluation of the dietary subject’s nutritional value and nutrient balance may prove significant in corroborating the findings.

It is also recommended that individuals with a potentially low antioxidant capacity of the body and an increased risk of oxidative stress integrate antioxidant-dense snacks into their balanced diet. Further studies should be conducted on a larger population and among patients with other conditions, and should include the seasonality of consumption.

## Figures and Tables

**Table 1:** Baseline sample characteristics by low (LowA)- and high (HighA)-antioxidant-capacity profile ^1^.

Characteristics	Total Sample	Antioxidant Capacity Profile		*p*-Value
		LowA	HighA	
Sample number	48	22	26	
Age, years	59.7 ± 2.0	59.5 ± 14.7	59.9 ± 9.6	0.90
Male, N (%)	21 (44)	8 (36)	13 (50)	0.34
Urban residence, N (%)	42 (88)	18 (82)	24 (92)	0.39
Higher education, N (%)	7 (14)	0 (0)	7 (27)	0.01
Physically active ^2^, N (%)	23 (48)	10 (45)	13 (5)	0.75
Current smokers, N (%)	13 (27)	7(32)	4 (15)	0.30
Hypertension, N (%)	10 (21)	6 (27)	4 (15)	0.47
Hyperlipidaemia, N (%)	28 (58)	10 (45)	18 (69)	0.10
Diabetes mellitus, N (%)	11 (23)	4 (18)	7 (27)	0.51
BMI (kg/m^2^)	30.5 ± 4.5	31.3 ± 4.7	29.9 ± 4.3	0.28
Obesity ^3^, N (%)	21 (44)	12 (55)	9 (35)	0.24
WC (cm)	105.0 ± 11.8	105.2 ± 12.2	104.2 ± 11.7	0.86
Antithrombotic agents ^4^, N (%)	4 (8)	3 (17)	1 (4)	0.32
BP-lowering drugs ^5^, N (%)	4 (8)	3 (17)	1 (4)	0.32
Lipid-modifying drugs ^6^, N (%)	5 (8)	4 (18)	1 (4)	0.16

^1^ Data presented as N (sample percentage in %) or mean ± standard deviation; ^2^ self-reported; ^3^ defined as BMI ≥ 30 kg/m^2^; ^4^ antithrombotic agents: ATC code B01; ^5^ blood-pressure-lowering drugs: ATC codes C02, C03, C07, C08, and C09; ^6^ lipid-modifying drugs: ATC code C10; *p*-value for difference between groups was obtained using Chi-square or Fisher’s exact test for categorical variables and *t*-test for continuous variables.

**Table 2:** Mean changes in baseline and blood and urinary markers after dietary intervention according to low (LowA)- and high (HighA)-antioxidant-capacity profile ^1^.

Variables	Total Sample		Antioxidant Capacity Profile				Difference in Between-Group Change ^3^
			LowA		HighA		
	Baseline	Change ^2^	Baseline	Change ^2^	Baseline	Change ^2^	
Sample number	48	48	22	22	26	26	22/26
**Lipid profile**							
HDL (mg/dL)	55 (51; 60)	0% (−4%; 4%)	55 (50; 61)	1% (−7%; 9%)	56 (49; 62)	−1% (−6%; 3%)	−211% (−965%; 544%)
LDL (mg/dL)	121 (108; 133)	−5% (−12%; 2%)	108 (93; 124)	−8% (−17%; 1%)	131 (112; 149)	−3% (−13%; 7%)	−55% (−159%; 49%)
HDL/LDL Ratio	0.51 (0.45; 0.57)	6% (−4%; 16%)	0.55 (0.48; 0.62)	13% (−3%; 29%)	0.48 (0.38; 0.58)	−1% (−14%; 13%)	−105% (−222%; 13%)
Cholesterol (mg/dL)	205 (190; 220)	−4% (−9%; 1%)	187 (167; 207)	−5% (−13%; 2%)	220 (198; 242)	−3% (−10%; 4%)	−35% (−168%; 97%)
Triglycerides (mg/dL)	135 (118; 151)	1% (−8%; 10%)	116 (94; 138)	−7% (−21%; 7%)	150 (127; 173)	7% (−5%; 19%)	−228% (−418%; −38%)
**Oxidative stress markers**							
F2-IsoP (ng/mg creatinine)	2.63 (2.08; 3.33)	−1% (−19%; 22%)	1.96 (1.61; 2.38)	29% (16%; 44%)	3.38 (2.28; 4.99)	−21% (−38%; 2%)	−38% * (−58%; −11%)
Urine F2-IsoP (ng/mL)	2.62 (1.88; 3.65)	−17% (−38%; 10%)	1.72 (1.27; 2.33)	18% (−2%; 41%)	3.74 (2.19; 6.39)	−39% *** (−56%; −15%)	−48% * (−69%; −13%)
OxLDL (μg/mL)	1.11 (0.79; 1.57)	−24% ** (−37%; −7%)	1.11 (0.61; 1.99)	−20% (−37%; −4%)	1.12 (0.72; 1.74)	−27% * (−41%; −8%)	−8% (−39%; 37%)
OxLDL/LDL ratio	0.01 (0.007; 0.014)	−19% * (−34%; 0%)	0.01 (0.01; 0.02)	−12% (−26%; 5%)	0.01 (0.01; 0.01)	−24% * (−31%; −3%)	−14% (−43%; 30%)
Uric acid (UA) (mg/dL)	5.6 (5.2; 6)	6% * (1%; 11%)	5.4 (4.7; 6)	5% (−3%; 13%)	5.8 (5.2; 6.4)	6% (0%; 12%)	31% (−174%; 237%)
**Antioxidant capacity markers**							
α-Tocopherol (μg/mL serum)	14.9 (14; 15.8)	4% (−2%; 10%)	12.4 (11.2; 13.5)	17% *** (8%; 26%)	17.0 (16.3; 17.8)	−4% (−10%; 3%)	−131% *** (−204%; −59%)
β-Carotene (μg/mL serum)	0.95 (0.85; 1.05)	23% *** (14%; 31%)	0.96 (0.77; 1.14)	23% *** (10%; 37%)	0.94 (0.82; 1.05)	22% *** (11%; 33%)	−8% (−78%; 62%)
Retinol (μg/mL serum)	2.7 (2.5; 2.8)	7% *** (3%; 11%)	2.39 (2.27; 2.51)	11% *** (3%; 18%)	2.89 (2.74; 3.05)	4% (−1%; 9%)	−56% (−141%; 28%)
**Inflammatory markers**							
IL-6 (pg/mL)	4.6 (3.2; 6.7)	−50% *** (−40%; −70%)	4 (2.7; 5.9)	−40% * (−50%; −70%)	5.2 (2.7; 9.9)	−50% *** (−70%; −30%)	−22% (−58%; 36%)
Hs-CRP (mg/L)	1.7 (1.3; 2.3)	0% (−20%;30%)	1.8 (1.2; 2.6)	30% (−10%; 70%)	1.7 (1.1; 2.6)	10% (−20%; 0%)	−29% (−56%; 27%)

^1^ Values presented as means (with 95% confidence interval), except urine F2-isoprostanes, OxLDL, OxLDL/LDL ratio, IL-6, and hs-CRP, which are presented as geometric means (with 95% confidence interval) due to non-normal distribution. ^2^ Within-group differences presented as % (with 95% confidence interval) and calculated as (Change-Baseline) × 100/Baseline, Change = (After DI − Baseline) × 100/Baseline, except for non-normally distributed variables which are presented as % geometric mean ratio (with 95% confidence interval) and calculated as (Change/Baseline). Within-group differences were tested using a paired *t*-test, adjusted for sex, age, BMI, and Q-ORAC. Statistical significance: * *p* < 0.5, ** *p* < 0.01, *** *p* < 0.001. ^3^ Between-group differences over time presented as % (with 95% confidence interval) and calculated as (HighA − LowA) × 100/LowA, except for non-normally distributed variables, which are presented as % geometric mean ratio (with 95% confidence interval) and calculated as (HighA/LowA). Between-group differences over time were tested using a linear mixed model adjusted for sex, age, BMI, and Q-ORAC. Statistical significance: * *p* < 0.5, ** *p* < 0.01, *** *p* < 0.001.

**Table 3:** Mean baseline and changes in dietary intake (per day) after DI according to low (LowA)- and high (HighA)-antioxidant-capacity profile.

Dietary Intake	Total Sample ^1^		LowA		HighA		Difference in Between-Group Change ^3^
	Baseline	Change ^2^	Baseline	Change ^2^	Baseline	Change ^2^	
Energy (kcal)	1654 (1517; 1792)	−6% (−17%; 6%)	1741 (1522; 1959)	−15% (−31%; 0%)	1581 (1397; 1766)	3% (−14%; 21%)	−120% (−258%; 18%)
Carbohydrates (%E)	43 (40.4; 45.6)	8% ** (4%; 13%)	45.1 (41.3; 48.9)	1% (−6%; 8%)	41.3 (37.6; 45)	15% *** (9%; 22%)	1283% *** (423%; 2142%)
Protein (%E)	17.6 (16,7; 18.5)	−2% (−9%; 5%)	16,9 (15,8; 18)	3% (−8%; 15%)	18.2 (16.8; 19.6)	−7% (−16%; 2%)	−313% (−743%; 117%)
Fat (%E)	39.4 (36.4; 42.3)	−4% (−11%; 2%)	38 (33.6; 42.5)	−3% (−13%; 7%)	40.5 (36.3; 44.7)	−6% (−16%; 4%)	130% (−384%; 645%)
Cholesterol (mg)	272 (243; 302)	−15% ** (−25%; −4%)	255 (212; 298)	−12% (−29%; 5%)	287 (244; 329)	−17% (−31%; −2%)	56% (−136%; 247%)
SFA (g)	25.8 (22.2; 29.4)	−20% ** (−36%; −5%)	27.6 (21.4; 33.9)	−25% (−47%; −3%)	24.3 (19.8; 28.8)	−16% (−38%; 6%)	−43% (−157%; 71%)
SFA (%E)	13.8 (12.6; 15.1)	−15% *** (−24%; −7%)	13.9 (12.1; 15.7)	−11% (−24%; 1%)	13.8 (12; 15.5)	−19% * (−31%; −7%)	65% (−82%; 212%)
Fibre (g)	22 (18.7; 25.3)	−7% (−24%; 10%)	24 (19.9; 28.2)	−21% (−44%; 2%)	20,3 (15.2; 25.5)	7% (−19%; 33%)	−129% (−275%; 17%)
T-ORAC (µmolTE/day)	22,674 (19,135; 26,213)	56% *** (32%; 79%)	23,991 (19,772; 28,209)	37% ** (6%; 67%)	21,560 (15,843; 27,277)	73% *** (37%; 109%)	80% (−38%; 197%;)
Q-ORAC (µmolTE/1000 kcal)	13,760 (11,989; 15,531)	68% *** (51%; 86%)	14,458 (11,744; 17,172)	58% *** (34%; 83%)	13,170 (10,690; 15,650)	77% *** (51%; 104%)	20% (−37%; 78%)
Total phenolics (mg)	1590 (1227; 1953)	−5% (−30%; 21%)	1731 (1297; 2165)	−18% (−49%; 14%)	1471 (885; 2057)	8% (−34%; 51%)	−140% (−404%; 123%)
Total flavonoids (mg)	810 (699; 920)	10% (−11%; 31%)	781 (624; 938)	7% (−23%; 37%)	834 (669; 999)	13% (−18%; 44%)	85% (−499%; 669%)
Flavan-3-ol (mg)	660 (557; 763)	1% (−21%; 23%)	622 (489; 754)	2% (−27%; 32%)	693 (533; 854)	−1% (−34%; 32%)	−131% (−2068%; 1806%)
Flavanones (mg)	25.6 (17.9; 33.3)	−54% ** (−85%; −22%)	28.5 (14; 42.9)	−70% *** (−114%; −25%)	23.1 (14.9; 31.4)	−38% (−84%; 8%)	−56% (−137%; 25%)
Flavones	221 (99; 344)	−16% (−63%; 30%)	273 (16; 529)	−33% (−105%; 40%)	178 (86; 269)	5% (−54%; 64%)	−110% (−352%; 133%)
Total proanthocyanidins	271 (203; 339)	−17% (−46%; 12%)	265 (189; 342)	−12% (−46%; 22%)	276 (163; 389)	−21% (−68%; 26%)	86% (−399%; 572%)
Vitamin A	1125 (877; 1373)	−12% (−35%; 11%)	1057 (912; 1203)	−9% (−32%; 14%)	1183 (727; 1639)	−14% (−52%; 23%)	75% (−439%; 589%)
β-Carotene (µg)	5306 (3468; 7143)	−11% (−45%; 22%)	4804 (3651; 5956)	−9% (−41%; 23%)	5730 (2371; 9090)	−13% (−69%; 42%)	75% (−714%; 865%)
Vitamin E (mg)	9.1 (7.9; 10.2)	2% (−12%; 16%)	9.1 (7.6; 10.7)	−10% (−29%; 9%)	9 (7.3; 10.8)	12% (−9%; 32%)	−216% (−489%; 58%)
Vitamin C (mg)	85.4 (65.2; 105.7)	−27% * (−51%; −3%)	89 (68; 110)	−29% (−55%; −2%)	82 (48; 117)	−26% (−66%; 14%)	−18% (−171%; 136%)

^1^ Values presented as means (with 95% confidence interval); ^2^ within-group differences presented as % (with 95% confidence interval) and calculated as (Change-Baseline) × 100/Baseline; within-group differences were tested using a paired *t*-test, and between-group differences over time were tested using a linear mixed model adjusted for sex, age, BMI, and Q-ORAC. Statistical significance: * *p* < 0.5, ** *p* < 0.01, *** *p* < 0.001. ^3^ Between-group differences over time presented as % (with 95% confidence interval) and calculated as (HighA − LowA) × 100/LowA, except for non-normally distributed variables, which are presented as % geometric mean ratio (with 95% confidence interval) and calculated as (HighA/LowA). Statistical significance: * *p* < 0.5, ** *p* < 0.01, *** *p* < 0.001.

## Data Availability

Data are available upon request from the authors of the work due to ethical reason.

## Supplementary Materials

The following supporting information can be downloaded at https://www.mdpi.com/article/10.3390/nu17050806/s1: Table S1: Composition of LowA and HighA groups based on k-means clustering of serum antioxidant capacity markers. Table S2: Indexes of antioxidant capacity of the food set consumed during the dietary intervention.

## References

[1] Dhalla N.S., Temsah R.M., Netticadan T. (2000). Role of Oxidative Stress in Cardiovascular Diseases. J. Hypertens..

[2] Lindstrom M., DeCleene N., Dorsey H., Fuster V., Johnson C.O., LeGrand K.E., Mensah G.A., Razo C., Stark B., Turco J.V. (2022). Global Burden of Cardiovascular Diseases and Risks Collaboration, 1990–2021. J. Am. Coll. Cardiol..

[3] Institute for Health Metrics and Evaluation GBD Compare. https://vizhub.healthdata.org/gbd-compare/#.

[4] Perez-Vizcaino F., Duarte J. (2010). Flavonols and Cardiovascular Disease. Mol. Asp. Med..

[5] Siti H.N., Kamisah Y., Kamsiah J. (2015). The Role of Oxidative Stress, Antioxidants and Vascular Inflammation in Cardiovascular Disease (a Review). Vasc. Pharmacol..

[6] Stocker R. (2004). Role of Oxidative Modifications in Atherosclerosis. Physiol. Rev..

[7] Elahi M.M., Kong Y.X., Matata B.M. (2009). Oxidative Stress as a Mediator of Cardiovascular Disease. Oxid. Med. Cell Longev..

[8] Sugamura K., Keaney J.F. (2011). Reactive Oxygen Species in Cardiovascular Disease. Free Radic. Biol. Med..

[9] Madamanchi N.R., Vendrov A., Runge M.S. (2005). Oxidative Stress and Vascular Disease. Arterioscler. Thromb. Vasc. Biol..

[10] Csányi G., Miller F.J. (2014). Oxidative Stress in Cardiovascular Disease. Int. J. Mol. Sci..

[11] Senoner T., Dichtl W. (2019). Oxidative Stress in Cardiovascular Diseases: Still a Therapeutic Target?. Nutrients.

[12] Scioli M.G., Storti G., D’amico F., Guzmán R.R., Centofanti F., Doldo E., Miranda E.M.C., Orlandi A. (2020). Oxidative Stress and New Pathogenetic Mechanisms in Endothelial Dysfunction: Potential Diagnostic Biomarkers and Therapeutic Targets. J. Clin. Med..

[13] Rankin J.W., Andreae M.C., Oliver Chen C.Y., O’Keefe S.F. (2008). Effect of Raisin Consumption on Oxidative Stress and Inflammation in Obesity. Diabetes Obes. Metab..

[14] Dow C.A., Wertheim B.C., Patil B.S., Thomson C.A. (2013). Daily Consumption of Grapefruit for 6 Weeks Reduces Urine F2-Isoprostanes in Overweight Adults with High Baseline Values but Has No Effect on Plasma High-Sensitivity C-Reactive Protein or Soluble Vascular Cellular Adhesion Molecule 1. J. Nutr..

[15] Annuzzi G., Bozzetto L., Costabile G., Giacco R., Mangione A., Anniballi G., Vitale M., Vetrani C., Cipriano P., Della Corte G. (2014). Diets Naturally Rich in Polyphenols Improve Fasting and Postprandial Dyslipidemia and Reduce Oxidative Stress: A Randomized Controlled Trial. Am. J. Clin. Nutr..

[16] Billingsley H.E., Carbone S. (2018). The Antioxidant Potential of the Mediterranean Diet in Patients at High Cardiovascular Risk: An in-Depth Review of the PREDIMED. Nutr. Diabetes.

[17] Varadharaj S., Kelly O.J., Khayat R.N., Kumar P.S., Ahmed N., Zweier J.L. (2017). Role of Dietary Antioxidants in the Preservation of Vascular Function and the Modulation of Health and Disease. Front. Cardiovasc. Med..

[18] Rubín-García M., Vitelli-Storelli F., Álvarez-Álvarez L., Fitó M., Vázquez-Ruiz Z., Salas-Salvadó J., Corella D., Serra-Majem L., Warnberg J., Romaguera D. (2024). Prospective Association of Changes in (Poly)Phenol Intake, Body Weight and Physical Activity with Inflammatory Profile. Nutr. Metab. Cardiovasc. Dis..

[19] Mulligan A.A., Lentjes M.A.H., Skinner J., Welch A.A. (2024). The Dietary Inflammatory Index and Its Associations with Biomarkers of Nutrients with Antioxidant Potential, a Biomarker of Inflammation and Multiple Long-Term Conditions. Antioxidants.

[20] Casas R., Sacanella E., Urpí-Sardà M., Chiva-Blanch G., Ros E., Martínez-González M.A., Covas M.I., Lamuela-Raventos R.M., Salas-Salvadó J., Fiol M. (2014). The Effects of the Mediterranean Diet on Biomarkers of Vascular Wall Inflammation and Plaque Vulnerability in Subjects with High Risk for Cardiovascular Disease. A Randomized Trial. PLoS ONE.

[21] Neto C.C. (2007). Cranberry and Blueberry: Evidence for Protective Effects against Cancer and Vascular Diseases. Mol. Nutr. Food Res..

[22] Curin Y., Andriantsitohaina R. (2005). Polyphenols as Potential Therapeutical Agents against Cardiovascular Diseases. Pharmacol. Rep..

[23] Serino A., Salazar G. (2018). Protective Role of Polyphenols against Vascular Inflammation, Aging and Cardiovascular Disease. Nutrients.

[24] Habauzit V., Morand C. (2012). Evidence for a Protective Effect of Polyphenols-Containing Foods on Cardiovascular Health: An Update for Clinicians. Ther. Adv. Chronic Dis..

[25] Kuriyama S., Shimazu T., Ohmori K., Kikuchi N., Nakaya N., Nishino Y., Tsubono Y., Tsuji I. (2006). Green Tea Consumption and Mortality Due to Cardiovascular Disease, Cancer, and All Causes in Japan: The Ohsaki Study. JAMA.

[26] Pang J., Zhang Z., Zheng T., Yang Y.J., Li N., Bai M., Peng Y., Zhang J., Li Q., Zhang B. (2015). Association of Green Tea Consumption with Risk of Coronary Heart Disease in Chinese Population. Int. J. Cardiol..

[27] Wang Q.-M., Gong Q.-Y., Yan J.-J., Zhu J.-, Tang J.-J., Wang M.-W., Yang Z.-J., Wang L.-S. (2010). Association Between Green Tea Intake and Coronary Artery Disease in a Chinese Population. Circ. J..

[28] Li X., Yu C., Guo Y., Bian Z., Si J., Yang L., Chen Y., Ren X., Jiang G., Chen J. (2017). Tea Consumption and Risk of Ischaemic Heart Disease. Heart.

[29] Draijer R., de Graaf Y., Slettenaar M., de Groot E., Wright C.I. (2015). Consumption of a Polyphenol-Rich Grape-Wine Extract Lowers Ambulatory Blood Pressure in Mildly Hypertensive Subjects. Nutrients.

[30] Lee A., Thurnham D.I., Chopra M. (2000). Consumption of Tomato Products with Olive Oil but Not Sunflower Oil Increases the Antioxidant Activity of Plasma. Free Radic. Biol. Med..

[31] Razavi S.M., Gholamin S., Eskandari A., Mohsenian N., Ghorbanihaghjo A., Delazar A., Rashtchizadeh N., Keshtkar-Jahromi M., Argani H. (2013). Red Grape Seed Extract Improves Lipid Profiles and Decreases Oxidized Low-Density Lipoprotein in Patients with Mild Hyperlipidemia. J. Med. Food.

[32] Kujawska M., Ewertowska M., Adamska T., Ignatowicz E., Gramza-Michałowska A., Jodynis-Liebert J. (2016). Protective Effect of Yellow Tea Extract on *N*-Nitrosodiethylamine-Induced Liver Carcinogenesis. Pharm. Biol..

[33] Czlapka-Matyasik M., Gut P. (2023). A Preliminary Study Investigating the Effects of Elevated Antioxidant Capacity of Daily Snacks on the Body’s Antioxidant Defences in Patients with CVD. Appl. Sci..

[34] Czlapka-Matyasik M., Gramza-Michalowska A. (2023). The Total Dietary Antioxidant Capacity, Its Seasonal Variability, and Dietary Sources in Cardiovascular Patients. Antioxidants.

[35] Redlich C.A., Chung J.S., Cullen M.R., Blaner W.S., Van Bennekum A.M., Berglund L. (1999). Effect of Long-Term Beta-Carotene and Vitamin A on Serum Cholesterol and Triglyceride Levels among Participants in the Carotene and Retinol Efficacy Trial (CARET). Atherosclerosis.

[36] Bastide N., Dartois L., Dyevre V., Dossus L., Fagherazzi G., Serafini M., Boutron-Ruault M.C. (2017). Dietary Antioxidant Capacity and All-Cause and Cause-Specific Mortality in the E3N/EPIC Cohort Study. Eur. J. Nutr..

[37] Mancini F.R., Affret A., Dow C., Balkau B., Bonnet F., Boutron-Ruault M.C., Fagherazzi G. (2018). Dietary Antioxidant Capacity and Risk of Type 2 Diabetes in the Large Prospective E3N-EPIC Cohort. Diabetologia.

[38] Henríquez-Sánchez P., Sánchez-Villegas A., Ruano-Rodríguez C., Gea A., Lamuela-Raventós R.M., Estruch R., Salas-Salvadó J., Covas M.I., Corella D., Schröder H. (2016). Dietary Total Antioxidant Capacity and Mortality in the PREDIMED Study. Eur. J. Nutr..

[39] Armas Díaz Y., Ferreiro Cotorruelo M.S., Battino M. (2023). The Role of Dietary Polyphenols in the Control of Chronic Noncommunicable Diseases. Food Saf. Health.

[40] Aryaeian N., Sedehi S.K., Arablou T. (2017). Polyphenols and Their Effects on Diabetes Management: A Review. Med. J. Islam. Repub. Iran.

[41] Kayama Y., Raaz U., Jagger A., Adam M., Schellinger I.N., Sakamoto M., Suzuki H., Toyama K., Spin J.M., Tsao P.S. (2015). Diabetic Cardiovascular Disease Induced by Oxidative Stress. Int. J. Mol. Sci..

[42] Gibson R.S. (2005). Principles Of Nutritional Assessment.

[43] Sobas K., Wadolowska L., Slowinska M.A., Czlapka-Matyasik M., Wuenstel J., Niedzwiedzka E. (2015). Like Mother, Like Daughter? Dietary and Non-Dietary Bone Fracture Risk Factors in Mothers and Their Daughters. Iran. J. Public Health.

[44] Szponar L., Rychlik E., Wolnicka K. (2000). Album of Photographs of Food Products and Dishes.

[45] Cao G., Prior R.L. (1998). Measurement of Oxygen Radical Absorbance Capacity in Biological Samples. Methods Enzym..

[46] Ou B.X., Hampsch-Woodill M., Prior R.L. (2001). Development and Validation of an Improved Oxygen Radical Absorbance Capacity Assay Using Fluorescein as the Fluorescent Probe. J. Agric. Food Chem..

[47] Haytowitz D.B., Bhagwat S. (2010). USDA Database for the Oxygen Radical Absorbance Capacity (ORAC) of Selected Foods.

[48] Kunachowicz H., Nadolna I., Przygoda B. (2015). Food Composition Tables.

[49] Dupuy A.M., Badiou S., Descomps B., Cristol J.P. (2003). Immunoturbidimetric Determination of C-Reactive Protein (CRP) and High-Sensitivity CRP on Heparin Plasma. Comparison with Serum Determination. Clin. Chem. Lab. Med..

[50] Milne G.L., Sanchez S.C., Musiek E.S., Morrow J.D. (2007). Quantification of F2-Isoprostanes as a Biomarker of Oxidative Stress. Nat. Protoc..

[51] Levey A.S., Eckardt K.U., Tsukamoto Y., Levin A., Coresh J., Rossert J., De Z.D., Hostetter T.H., Lameire N., Eknoyan G. (2005). Definition and Classification of Chronic Kidney Disease: A Position Statement from Kidney Disease: Improving Global Outcomes (KDIGO). Kidney Int..

[52] Belo L., Caslake M., Santos-Silva A., Castro E.M.B., Pereira-Leite L., Quintanilha A., Rebelo I. (2004). LDL Size, Total Antioxidant Status and Oxidised LDL in Normal Human Pregnancy: A Longitudinal Study. Atherosclerosis.

[53] Macut D., Damjanovic S., Panidis D., Spanos N., Glisic B., Petakov M., Rousso D., Kourtis A., Bjekic J., Milic N. (2006). Oxidised Low-Density Lipoprotein Concentration—Early Marker of an Altered Lipid Metabolism in Young Women with PCOS. Eur. J. Endocrinol. Eur. Fed. Endocr. Soc..

[54] Fossati P., Prencipe L., Berti G. (1980). Use of 3,5-Dichloro-2-Hydroxybenzenesulfonic Acid/4-Aminophenazone Chromogenic System in Direct Enzymic Assay of Uric Acid in Serum and Urine. Clin. Chem..

[55] MacCrehan W.A., Schönberger E., Schonberger E. (1987). Determination of Retinol, Alpha-Tocopherol, and Beta-Carotene in Serum by Liquid Chromatography with Absorbance and Electrochemical Detection. Clin. Chem..

[56] Casal S., Macedo B., Oliveira M.B. (2001). Simultaneous Determination of Retinol, Beta-Carotene and Alpha-Tocopherol in Adipose Tissue by High-Performance Liquid Chromatography. J. Chromatogr. B Biomed. Sci. Appl..

[57] Chauveau-Duriot B., Doreau M., Nozière P., Graulet B. (2010). Simultaneous Quantification of Carotenoids, Retinol, and Tocopherols in Forages, Bovine Plasma, and Milk: Validation of a Novel UPLC Method. Anal. Bioanal. Chem..

[58] Chan D.W., Perlstein M. (1987). Immunoassay: A Practical Guide.

[59] Lira F.S., Panissa V.L.G., Julio U.F., Franchini E. (2015). Differences in Metabolic and Inflammatory Responses in Lower and Upper Body High-Intensity Intermittent Exercise. Eur. J. Appl. Physiol..

[60] Perrigue P.M., Silva M.E., Warden C.D., Feng N.L., Reid M.A., Mota D.J., Joseph L.P., Tian Y.I., Glackin C.A., Gutova M. (2015). The Histone Demethylase Jumonji Coordinates Cellular Senescence Including Secretion of Neural Stem Cell-Attracting Cytokines. Mol. Cancer Res..

[61] Yoshikawa T., Hata J., Sakata S., Nagata T., Hirakawa Y., Hirooka Y., Tsutsui H., Kitazono T., Ninomiya T. (2021). Serum High-Sensitivity C-Reactive Protein Levels and the Development of Atrial Fibrillation in a General Japanese Population―The Hisayama Study―. Circ. J..

[62] Aleksandrova K., Koelman L., Rodrigues C.E. (2021). Dietary Patterns and Biomarkers of Oxidative Stress and Inflammation: A Systematic Review of Observational and Intervention Studies. Redox Biol..

[63] Koelman L., Egea Rodrigues C., Aleksandrova K. (2021). Effects of Dietary Patterns on Biomarkers of Inflammation and Immune Responses: A Systematic Review and Meta-Analysis of Randomized Controlled Trials. Adv. Nutr..

[64] Prescha A., Zabłocka-Słowińska K., Płaczkowska S., Gorczyca D., Luczak A., Majewska M., Grajeta H. (2018). Diet Quality and Its Relationship with Antioxidant Status in Patients with Rheumatoid Arthritis. Oxid. Med. Cell Longev..

[65] Huang Y., Ni Y., Yu L., Shu L., Zhu Q., He X. (2024). Dietary Total Antioxidant Capacity and Risk of Stroke: A Systematic Review and Dose-Response Meta-Analysis of Observational Studies. Front. Nutr..

[66] Rautiainen S., Levitan E.B., Orsini N., Åkesson A., Morgenstern R., Mittleman M.A., Wolk A. (2012). Total Antioxidant Capacity from Diet and Risk of Myocardial Infarction: A Prospective Cohort of Women. Am. J. Med..

[67] Yang M., Chung S.J., Chung C.E., Kim D.O., Song W.O., Koo S.I., Chun O.K. (2011). Estimation of Total Antioxidant Capacity from Diet and Supplements in US Adults. Br. J. Nutr..

[68] Chun O.K., Floegel A., Chung S.J., Chung C.E., Song W.O., Koo S.I. (2010). Estimation of Antioxidant Intakes from Diet and Supplements in U.S. Adults. J. Nutr..

[69] Ha K., Liao L.M., Sinha R., Chun O.K. (2023). Dietary Total Antioxidant Capacity, a Diet Quality Index Predicting Mortality Risk in US Adults: Evidence from the NIH-AARP Diet and Health Study. Antioxidants.

[70] Isabelle M., Lee B.L., Lim M.T., Koh W.P., Huang D., Ong C.N. (2010). Antioxidant Activity and Profiles of Common Fruits in Singapore. Food Chem..

[71] Isabelle M., Lee B.L., Lim M.T., Koh W.P., Huang D., Ong C.N. (2010). Antioxidant Activity and Profiles of Common Vegetables in Singapore. Food Chem..

[72] Rautiainen S., Larsson S., Virtamo J., Wolk A. (2012). Total Antioxidant Capacity of Diet and Risk of Stroke. Stroke.

[73] Człapka-Matyasik M., Ast K. (2014). Total Antioxidant Capacity and Its Dietary Sources and Seasonal Variability in Diets of Women with Different Physical Activity Levels. Pol. J. Food Nutr. Sci..

[74] Prior R.L., Wu X. (2013). Diet Antioxidant Capacity: Relationships to Oxidative Stress and Health. Am. J. Biomed. Sci..

[75] Prior R.L. (2015). Oxygen Radical Absorbance Capacity (ORAC): New Horizons in Relating Dietary Antioxidants/Bioactives and Health Benefits. J. Funct. Foods.

[76] Bogard J.R., Downs S., Casey E., Farrell P., Gupta A., Miachon L., Naughton S., Staromiejska W., Reeve E. (2024). Convenience as a Dimension of Food Environments: A Systematic Scoping Review of Its Definition and Measurement. Appetite.

[77] Nakano S., Washizu A. (2020). Aiming for Better Use of Convenience Food: An Analysis Based on Meal Production Functions at Home. J. Health Popul. Nutr..

[78] Hu X., Dong D., Xia M., Yang Y., Wang J., Su J., Sun L., Yu H. (2020). Oxidative Stress and Antioxidant Capacity: Development and Prospects. New J. Chem..

[79] Tsimikas S. (2008). In Vivo Markers of Oxidative Stress and Therapeutic Interventions. Am. J. Cardiol..

[80] Strobel N.A., Fassett R.G., Marsh S.A., Coombes J.S. (2011). Oxidative Stress Biomarkers as Predictors of Cardiovascular Disease. Int. J. Cardiol..

[81] Martínez-Tomás R., Larqué E., González-Silvera D., Sánchez-Campillo M., Burgos M.I., Wellner A., Parra S., Bialek L., Alminger M., Pérez-Llamas F. (2012). Effect of the Consumption of a Fruit and Vegetable Soup with High in Vitro Carotenoid Bioaccessibility on Serum Carotenoid Concentrations and Markers of Oxidative Stress in Young Men. Eur. J. Nutr..

[82] Cao G.H., Booth S.L., Sadowski J.A., Prior R.L. (1998). Increases in Human Plasma Antioxidant Capacity after Consumption of Controlled Diets High in Fruit and Vegetables. Am. J. Clin. Nutr..

[83] Tesoriere L., Butera D., Pintaudi A.M., Allegra M., Livrea M.A. (2004). Supplementation with Cactus Pear (Opuntia Ficus-Indica) Fruit Decreases Oxidative Stress in Healthy Humans: A Comparative Study with Vitamin C. Am. J. Clin. Nutr..

[84] van ’t Erve T.J., Kadiiska M.B., London S.J., Mason R.P. (2017). Classifying Oxidative Stress by F2-Isoprostane Levels across Human Diseases: A Meta-Analysis. Redox Biol..

[85] van ‘t Erve T.J. (2018). Strategies to Decrease Oxidative Stress Biomarker Levels in Human Medical Conditions: A Meta-Analysis on 8-Iso-Prostaglandin F2α. Redox Biol..

[86] Meyer K.A., Sijtsma F.P.C., Nettleton J.A., Steffen L.M., Van Horn L., Shikany J.M., Gross M.D., Mursu J., Traber M.G., Jacobs D.R. (2012). Dietary Patterns Are Associated with Plasma F2-Isoprostanes in an Observational Cohort Study of Adults. Free Radic. Biol. Med..

[87] Kempf K., Herder C., Erlund I., Kolb H., Martin S., Carstensen M., Koenig W., Sundvall J., Bidel S., Kuha S. (2010). Effects of coffee consumption on subclinical inflammation and other risk factors for type 2 diabetes: A clinical trial. Am. J. Clin. Nutr..

[88] Ochiai R., Chikama A., Kataoka K., Tokimitsu I., Maekawa Y., Ohishi M., Rakugi H., Mikami H. (2009). Effects of Hydroxyhydroquinone-Reduced Coffee on Vasoreactivity and Blood Pressure. Hypertens. Res..

[89] Petrosino T., Serafini M. (2014). Antioxidant Modulation of F2-Isoprostanes in Humans: A Systematic Review. Crit. Rev. Food Sci. Nutr..

[90] Gagliardi A.C.M., Miname M.H., Santos R.D. (2009). Uric Acid: A Marker of Increased Cardiovascular Risk. Atherosclerosis.

[91] Kanbay M., Segal M., Afsar B., Kang D.-H., Rodriguez-Iturbe B., Johnson R.J. (2013). The Role of Uric Acid in the Pathogenesis of Human Cardiovascular Disease. Heart.

[92] Sautin Y.Y., Johnson R.J. (2008). Uric Acid: The Oxidant-Antioxidant Paradox. Nucleosides Nucleotides Nucleic Acids.

[93] Ames B.N., Cathcart R., Schwiers E., Hochstein P. (1981). Uric Acid Provides an Antioxidant Defense in Humans against Oxidant- and Radical-Caused Aging and Cancer: A Hypothesis. Proc. Natl. Acad. Sci. USA.

[94] Simic M.G., Jovanovic S.V. (1989). Antioxidation Mechanisms of Uric Acid. J. Am. Chem. Soc..

[95] Becker B.F. (1993). Towards the Physiological Function of Uric Acid. Free Radic. Biol. Med..

[96] Strazzullo P., Puig J.G. (2007). Uric Acid and Oxidative Stress: Relative Impact on Cardiovascular Risk?. Nutr. Metab. Cardiovasc. Dis..

[97] Hellsten Y., Tullson P.C., Richter E.A., Bangsbo J. (1997). Oxidation of Urate in Human Skeletal Muscle during Exercise. Free Radic. Biol. Med..

[98] Kostka-Jeziorny K., Tykarski A. (2009). Losartan, Allopurinol—Czy Są Dowody, Że Hiperurykemia Może Stać Siȩ Kolejnym Celem Terapii w Prewencji Ryzyka Sercowo-Naczyniowego u Pacjentów z Nadciśnieniem Tȩtniczym?. Nadcisnienie Tetnicze.

[99] Samborski P., Bogdański P., Pupek-Musialik D. Nowe Spojrzenie Na Kwas Moczowy u Chorych z Zespołem Metabolicznym—Fakty i Kontrowersje. https://journals.viamedica.pl/eoizpm/article/view/26041/20851.

[100] Hayden M.R., Tyagi S.C. (2004). Uric Acid: A New Look at an Old Risk Marker for Cardiovascular Disease, Metabolic Syndrome, and Type 2 Diabetes Mellitus: The Urate Redox Shuttle. Nutr. Metab..

[101] Hellsten Y., Sjodin B., Richter E. (1998). Urate Uptake and Lowered ATP Levels in Human Muscle after High-Intensity Intermittent Exercise. Am. J. Physiol..

[102] Kang D.H., Ha S.K. (2014). Uric Acid Puzzle: Dual Role as Anti-Oxidantand pro-Oxidant. Electrolyte Blood Press..

[103] Fernandez-Calle P., Molina J.A., Jimenez-Jimenez F.J., Vazquez A., Pondal M., Garcia-Ruiz P.J., Urra D.G., Domingo J., Codoceo R. (1992). Serum Levels of Alpha-Tocopherol (Vitamin E) in Parkinson’s Disease. Neurology.

[104] Leotsinidis M., Alexopoulos A., Schinas V., Kardara M., Kondakis X. Plasma Retinol and Tocopherol Levels in Greek Elderly Population from an Urban and a Rural Area: Associations with the Dietary Habits. http://han.up.poznan.pl/han/webofknowledgehh/download.springer.com/static/pdf/430/art%253A10.1023%252FA%253A1010895227352.pdf?originUrl=http%253A%252F%252Flink.springer.com%252Farticle%252F10.1023%252FA%253A1010895227352&token2=exp=1455750966~acl=%252Fstatic%252Fpdf%252F430%25.

[105] Bahcecioglu I.H., Yalniz M., Ilhan N., Ataseven H., Ozercan I.H. (2005). Levels of Serum Vitamin A, Alpha-Tocopherol and Malondialdehyde in Patients with Non-Alcoholic Steatohepatitis: Relationship with Histopathologic Severity. Int. J. Clin. Pr..

[106] Nagamma T., Baxi J., Singh P.P. (2014). Status of Oxidative Stress and Antioxidant Levels in Smokers with Breast Cancer from Western Nepal. Asian Pac. J. Cancer Prev..

[107] Aguiló A., Tauler P., Fuentespina E., Tur J.A., Córdova A., Pons A. (2005). Antioxidant Response to Oxidative Stress Induced by Exhaustive Exercise. Physiol. Behav..

[108] Gornicka M. (2013). Badania Nad Wpływem Alfa-Tokoferolu Na Homeostazę Redoks Organizmu Poddanego Wysiłkowi Fizycznemu. Ph.D. Thesis.

[109] Rietjens I.M.C.M., Boersma M.G., de Haan L., Spenkelink B., Awad H.M., Cnubben N.H.P.P., Van Zanden J.J., van der Woude H., Alink G.M., Koeman J.H. (2002). The Pro-Oxidant Chemistry of the Natural Antioxidants Vitamin C, Vitamin E, Carotenoids and Flavonoids. Environ. Toxicol. Pharmacol..

[110] Igielska-Kalwat J., Gościańska J., Nowak I. (2015). Karotenoidy Jako Naturalne Antyoksydanty. Postep. Hig. Med. Dosw..

[111] Sies H., Stahl W., Sundquist A.R. (1992). Antioxidant Functions of Vitamins. Vitamins E and C, Beta-Carotene, and Other Carotenoids. Ann. N. Y. Acad. Sci..

[112] Krinsky N.I., Landrum J.T., Bone R.A. (2003). Biologic Mechanisms of the Protective Role of Lutein and Zeaxanthin in the Eye. Annu. Rev. Nutr..

[113] Blot W.J., Li J.Y., Taylor P.R., Guo W., Dawsey S., Wang G.Q., Yang C.S., Zheng S.F., Gail M., Li G.Y. (1993). Nutrition Intervention Trials in Linxian, China: Supplementation with Specific Vitamin/Mineral Combinations, Cancer Incidence, and Disease-Specific Mortality in the General Population. J. Natl. Cancer Inst..

[114] Abrego-Guandique D.M., Bonet M.L., Caroleo M.C., Cannataro R., Tucci P., Ribot J., Cione E. (2023). The Effect of Beta-Carotene on Cognitive Function: A Systematic Review. Brain Sci..

[115] Fiedor J., Burda K. (2014). Potential Role of Carotenoids as Antioxidants in Human Health and Disease. Nutrients.

[116] Gale C.R., Hall N.F., Phillips D.I., Martyn C.N. (2001). Plasma Antioxidant Vitamins and Carotenoids and Age-Related Cataract. Ophthalmology.

[117] Jansen E., Ruskovska T. (2015). Serum Biomarkers of (Anti)Oxidant Status for Epidemiological Studies. Int. J. Mol. Sci..

[118] Kritchevsky S.B. (1999). β-Carotene, Carotenoids and the Prevention of Coronary Heart Disease. J. Nutr..

[119] Tavani A., La Vecchia C. (1999). Beta-Carotene and Risk of Coronary Heart Disease. A Review of Observational and Intervention Studies. Biomed. Pharmacother..

[120] Yeşilbursa D., Serdar Z., Serdar A., Dirican M., Gemici K., Türel B., Aydin A., Cordan J. (2000). Oxidative Stress in Patients with Chronic Heart Failure. Heart.

[121] Garcia A.L., Mohan R., Koebnick C., Bub A., Heuer T., Strassner C., Groeneveld M.J., Katz N., Elmadfa I., Leitzmann C. (2010). Plasma Beta-Carotene Is Not a Suitable Biomarker of Fruit and Vegetable Intake in German Subjects with a Long-Term High Consumption of Fruits and Vegetables. Ann. Nutr. Metab..

[122] Nogueira C., Borges F., Lameu E., Franca C., Rosa C.L.d.S., Ramalho A. (2015). Retinol, β-Carotene and Oxidative Stress in Systemic Inflammatory Response Syndrome. Rev. Assoc. Med. Bras..

[123] Tébi A., Belbraouet S., Chau N., Debry G. (2000). Plasma Vitamin, β-Carotene, and α-Tocopherol Status According to Age and Disease in Hospitalized Elderly. Nutr. Res..

[124] Cooney R.V., Bertram J.S., Hankin J.H., Kolonel L.N., Miyake A., Billings K., Bourne W. (1991). Relationship between Dietary, Serum, and Tissue Levels of Carotenoids. Cancer Lett..

[125] Burri B.J., Neidlinger T.R., Clifford A.J. (2001). Serum Carotenoid Depletion Follows First-Order Kinetics in Healthy Adult Women Fed Naturally Low Carotenoid Diets. J. Nutr..

[126] Martínez-Tomás R., Pérez-Llamas F., Sánchez-Campillo M., González-Silvera D., Cascales A.I., García-Fernández M., López-Jiménez J.Á., Navarro S.Z., Burgos M.I., López-Azorín F. (2012). Daily Intake of Fruit and Vegetable Soups Processed in Different Ways Increases Human Serum β-Carotene and Lycopene Concentrations and Reduces Levels of Several Oxidative Stress Markers in Healthy Subjects. Food Chem..

[127] Omenn G.S., Goodman G.E., Thornquist M.D., Balmes J., Cullen M.R., Glass A., Keogh J.P., Meyskens F.L., Valanis B., Williams J.H. (1996). Risk Factors for Lung Cancer and for Intervention Effects in CARET, the Beta-Carotene and Retinol Efficacy Trial. J. Natl. Cancer Inst..

[128] Omenn G.S., Goodman G.E., Thornquist M.D., Balmes J., Cullen M.R., Glass A., Keogh J.P., Valanis B., Meyskens F.L., Williams J.H. (1996). Effects of a Combination of Beta Carotene and Vitamin A on Lung Cancer and Cardiovascular Disease. N. Engl. J. Med..

[129] Omenn G.S. (2007). Chemoprevention of Lung Cancers: Lessons from CARET, the Beta-Carotene and Retinol Efficacy Trial, and Prospects for the Future. Eur. J. Cancer Prev..

[130] Bellovino D., Apreda M., Gragnoli S., Massimi M., Gaetani S. (2003). Vitamin A Transport: In Vitro Models for the Study of RBP Secretion. Mol. Asp. Med..

[131] Kartha V.N., Krishnamurthy S. (1977). Antioxidant Function of Vitamin A. Int. J. Vitam. Nutr. Res..

[132] Livrea M.A., Tesoriere L. (1998). Antioxidant Activity of Vitamin A within Lipid Environments. Subcell. Biochem..

[133] Livrea M.A., Tesoriere L., Bongiorno A., Pintaudi A.M., Ciaccio M., Riccio A. (1995). Contribution of Vitamin A to the Oxidation Resistance of Human Low Density Lipoproteins. Free Radic. Biol. Med..

[134] Wald N.J., Boreham J., Hayward J.L., Bulbrook R.D. (1984). Plasma Retinol, Beta-Carotene and Vitamin E Levels in Relation to the Future Risk of Breast Cancer. Br. J. Cancer.

[135] Chang C.-Y., Chen J.-Y., Ke D., Hu M.-L. (2005). Plasma Levels of Lipophilic Antioxidant Vitamins in Acute Ischemic Stroke Patients: Correlation to Inflammation Markers and Neurological Deficits. Nutrition.

[136] Hermanowska-Szpakowicz T., Stankiewicz A., Kondrusik M., Zajkowska J. (2005). Stezenie Witaminy A, E Oraz C w Surowicy Krwi Osob Posiadajacych Przeciwciała Przeciwko Borrelia Burgdorferi—Bez Objawow Zakażenia—Doniesienie Wstepne. Przegląd Epidemilogiczny.

[137] Ruiz Rejón F., Martín-Peña G., Granado F., Ruiz-Galiana J., Blanco I., Olmedilla B. (2002). Plasma status of retinol, alpha- and gamma-tocopherols, and main carotenoids to first myocardial infarction: Case control and follow-up study. Nutrition.

[138] Mecocci P., Polidori M.C., Troiano L., Cherubini A., Cecchetti R., Pini G., Straatman M., Monti D., Stahl W., Sies H. (2000). Plasma Antioxidants and Longevity: A Study on Healthy Centenarians. Free Radic. Biol. Med..

[139] Liu Y., Wang D., Li D., Sun R., Xia M. (2014). Associations of Retinol-Binding Protein 4 with Oxidative Stress, Inflammatory Markers, and Metabolic Syndrome in a Middle-Aged and Elderly Chinese Population. Diabetol. Metab. Syndr..

[140] Xiang J., Nagaya T., Huang X.-E., Kuriki K., Imaeda N., Tokudome Y., Sato J., Fujiwara N., Maki S., Tokudome S. (2008). Sex and Seasonal Variations of Plasma Retinol, Alpha-Tocopherol, and Carotenoid Concentrations in Japanese Dietitians. Asian Pac. J. Cancer Prev..

