# Supplementary material for: Changes in Oxidative Stress, Inflammatory Markers, and Lipid Profile After a 6-Week High-Antioxidant-Capacity Dietary Intervention in CVD Patients
Source: Nutrients. 2025 Feb 26;17(5):806. doi: 10.3390/nu17050806 (PMC11902212; doi:10.3390/nu17050806)
Supplement: Supplementary file 1 [file nutrients-17-00806-s001.zip › nutrients-3431753-supplementary.pdf]

Suppl. Table S1. Composition of LowA and HighA groups based on K-means clustering of serum antioxidant capacity markers[1]

**Marker**	**LowA (N=22)**	**HighA (N=26)**	***P***
α-Tocopherol (µg/ml serum)	-0,79 (-1,15; -0,42)	0,67 (0,44; 0,89)	<0,001
β-Carotene (µg/ml serum)	0,03 (-0,5; 0,56)	-0,03 (-0,36; 0,31)	0,84
Retinol (µg/ml serum)	-0,65 (-0,94; -0,36)	0,55 (0,18; 0,92)	<0,001

[1]Data are presented as standardized means (Z-score) with a 95% CI. P value was obtained using a T-test.

Suppl. Table S2. Indexes of antioxidant capacity of the food set consumed during the dietary intervention

Foods	Average mass of the foods (g/week)	Indicators of the antioxidant capacity		
		T-ORAC[2] (µmolTE/week)	T-ORAC[3] (µmolTE/day)	Q-ORAC1000[4] (µmolTE/1000kcal)
Dark chocolate (90% of cocoa)	63,5	31 714	4 531	89 876
Strawberry crisps	45,1	21 467	3 067	15 467
Red wine *(cabernet sauvignon)*	302,8	13 696	1 957	66 458
Dried cranberries	86,7	7 881	1 126	15 857
Chokeberry-apple juice (100%)	406,0	7 825	1 118	131 211
Dried oregano	3,6	6 311	902	682 968
Walnuts	38,6	5 227	747	21 010
Dried thyme	3,2	5 036	719	521 342
Grated beetroot with horseradish	251,0	4 458	637	38 572
Hazelnuts	43,1	4 157	594	14 966
Prunes	39,0	3 143	449	29 501
Beetroot crisps	19,3	2 771	396	5 603
Green tea	1750	2 713	388	155 000
Dried apricots	73,2	2 367	338	11 383
Black pepper	6,4	2 179	311	137 762
Apple crisps[1]	48,7	2 081	297	1 706

Carrot crisps[1]	32,8	1 386	198	1 394
Tomato juice (100%)	401,5	1 951	279	34 671
Dried basil	2,9	1 771	253	272 016
Almonds	15,5	690	99	7 803
Tomato crisps[1]	4,4	464	66	1 984
Olive oil	62,3	232	33	422
Dried dill	4,1	180	26	160 779
Sum/mean	529	129 701	18 529	105 119

[1] Microwave dried product. [2] Total antioxidant capacities of foods consumed weekly. [3] Total antioxidant capacities of foods consumed daily. [4] Antioxidative density of foods consumed daily.
